# SIAH E3 UBIQUITIN LIGASE2 (SIAH2) DELETION MIMICS AGE-ASSOCIATED PHENOTYPES IN MOUSE MACROPHAGES

**DOI:** 10.1093/geroni/igad104.3591

**Published:** 2023-12-21

**Authors:** Bhaswati Ghosh, Pradip Panta, Matthew Scott, Elizabeth Floyd

**Affiliations:** Pennington Biomedical Research Center, Baton Rouge, Louisiana, United States; Southeastern University, Southeastern University, Louisiana, United States; Pennington Biomedical Research Center, Baton Rouge, Louisiana, United States; Pennington Biomedical Research Center, Baton Rouge, Louisiana, United States

## Abstract

Aging is generally characterized by physiological as well as metabolic decline. Crosstalk between metabolic organs is remarkably altered by aging. For one, adipose tissue exhibits distinct cellular and molecular attributes of aging. Impaired adipogenesis is one of the aging hallmarks. Previously, we demonstrated Siah E3 Ubiquitin Ligase2 (Siah2) is upregulated during adipogenesis. We are interested if and how Siah2 expression is altered by aging. Recently, the age-related decline in immune function was first detected in mouse adipose tissue. To explore the regulatory role of Siah2 in immune response, we generated mice with Siah2 knockout in macrophages (SKM) and observed elevated expression of inflammatory regulators including TNF-α and Trem2 in SKM macrophages compared to control. Additionally, we detected increased lipid accumulation and expression of Plin2 (lipid droplet marker) in the co-culture model of adipose tissue macrophages derived from SKM mice. These data are consistent with previous research showing an age-related increase in TNF-α expression and lipid content. Varying degrees of age-related alterations have been observed in different adipose tissue depots. For example, aging attenuates “beiging” of white adipose tissue (WAT) to brown adipose-like tissue and consequently is associated with lower levels of mitochondrial respiration. Interestingly, oxygen consumption rate, a quantitative measure of mitochondrial function, is lower in the SKM macrophages compared to the control group, hinting that Siah2 may regulate browning of WAT during aging. Altogether, these data suggest that Siah2 KO phenotypes mimic major aging hallmarks and that Siah2 could be a potential therapeutic target for age-related adipose tissue disorders.

